# Bis{6-bromo-4-chloro-2-[(*E*)-(2-chloro­phenyl)­imino­meth­yl]­phenolato-κ^2^ *N*,*O*}copper(II)

**DOI:** 10.1107/S1600536812025044

**Published:** 2012-07-25

**Authors:** Zhang Ping

**Affiliations:** a Department of Chemistry, Xianyang Normal University, Xianyang, Shaanxi 712000, People’s Republic of China

## Abstract

In the title compound, [Cu(C_13_H_7_BrCl_2_NO)_2_], or Cu*L*_2_ {where H*L*= 2-[(*E*)-(2-chloro­phenyl­imino)­meth­yl]-6-bromo-4-chloro­phenol}, the Cu^II^ atom is located on an inversion center and has a square-planar coordination. In the crystal, complex mol­ecules are linked via Cu⋯Cl inter­actions [2.9933 (11) Å], forming a two-dimensional network parallel to the *bc* plane. They are also Cl⋯Cl inter­actions [3.3709 (14) Å] present, which consolidate the two-dimensional network structure.

## Related literature

For applications and properties of bidentate Schiff base ligands and their metal complexes, see: Akine *et al.* (2002 ▶); Schuetz *et al.* (2004 ▶); Singh *et al.* (1997 ▶); Qi *et al.* (2007 ▶).
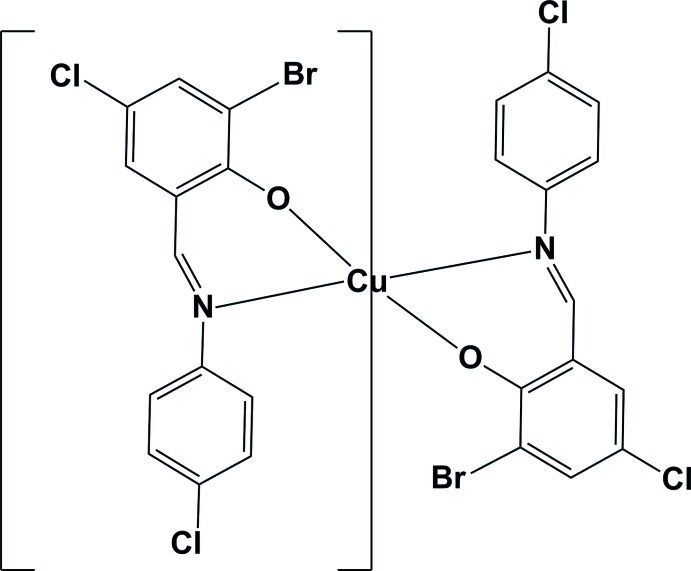


## Experimental

### 

#### Crystal data

[Cu(C_13_H_7_BrCl_2_NO)_2_]
*M* *_r_* = 751.55
Monoclinic, 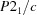

*a* = 11.064 (3) Å
*b* = 9.437 (2) Å
*c* = 13.277 (4) Å
β = 108.997 (3)°
*V* = 1310.8 (6) Å^3^
*Z* = 2
Mo *K*α radiation
μ = 4.32 mm^−1^
*T* = 153 K
0.46 × 0.42 × 0.42 mm

#### Data collection

Rigaku AFC10/Saturn724+ diffractometer
Absorption correction: multi-scan (*CrystalClear*; Rigaku, 2008 ▶) *T*_min_ = 0.242, *T*_max_ = 0.265
10994 measured reflections
3491 independent reflections
2777 reflections with *I* > 2σ(*I*)
*R*_int_ = 0.033

#### Refinement

*R*[*F* ^2^ > 2σ(*F* ^2^)] = 0.028
*wR*(*F* ^2^) = 0.065
*S* = 1.00
3491 reflections
169 parameters
H-atom parameters constrained
Δρ_max_ = 0.53 e Å^−3^
Δρ_min_ = −0.49 e Å^−3^

### 

Data collection: *CrystalClear* (Rigaku, 2008 ▶); cell refinement: *CrystalClear*; data reduction: *CrystalClear*; program(s) used to solve structure: *SHELXS97* (Sheldrick, 2008 ▶); program(s) used to refine structure: *SHELXL97* (Sheldrick, 2008 ▶); molecular graphics: *SHELXTL* (Sheldrick, 2008 ▶); software used to prepare material for publication: *SHELXTL*.

## Supplementary Material

Crystal structure: contains datablock(s) I, global. DOI: 10.1107/S1600536812025044/su2417sup1.cif

Structure factors: contains datablock(s) I. DOI: 10.1107/S1600536812025044/su2417Isup2.hkl

Additional supplementary materials:  crystallographic information; 3D view; checkCIF report

## Figures and Tables

**Fig. 1.**
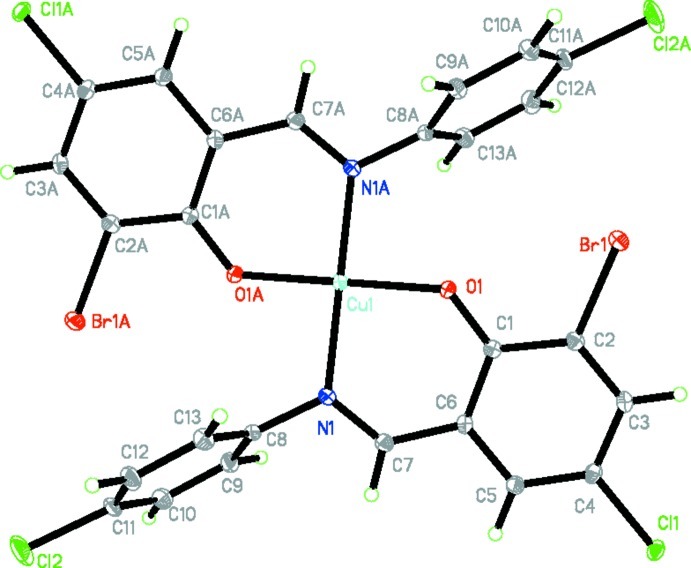
The molecular structure of the title compound, showing the numbering scheme. The displacement ellipsoids are drawn at the 30% probability level (symmetry code: (A) = -x + 1, -y + 1, -z + 1).

**Fig. 2.**
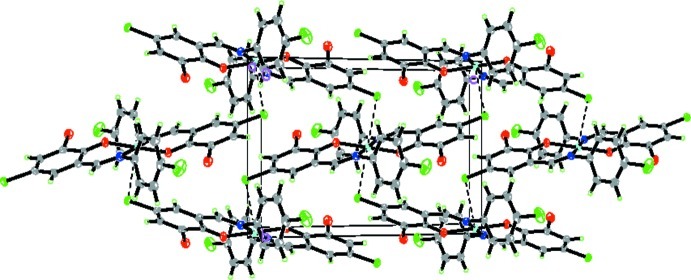
A view along the a axis of the crystal packing of the title compound. The Cu—Cl interactions leading to the formation of the two-dimensional network are shown as dashed lines.

## supplementary crystallographic information

### Comment

Bidentate Schiff base ligands of various types and their metal complexes have proved to be of significant interest in the areas of photoluminescence (Akine *et al.*, 2002), catalysis (Schuetz *et al.*, 2004), magnetism (Singh *et al.*, 1997) and molecular architectures (Qi *et al.*, 2007). The title complex was obtained from the reaction of 3-bromo-5-chlorosalicylaldehyde, 4-chlorobenzenamine and CuCl_2_.2H_2_O. We report herein on the synthesis and crystal structure of the title compound.

The molecular structure of the title complex, Cu*L*_2_, is shown in Fig. 1. The central Cu^II^ atom lies on an inversion center and has a square-planar coordination geometry, through the formation of two Cu—N and two Cu—O bonds with two bidentate 2-((*E*)-(2- chlorophenylimino)methyl)-6-bromo-4-chlorophenol (HL) ligands. The dihedral angle between the phenyl ring (C1—C6) and the chelate ring (O1/Cu1/N1/C7/C6/C1) is only 6.2() °. The two benzene rings in each ligand are inclined to one another by 65.97 (10)°. Bond angles also show that the coordination geometry about the copper atom is a slightly distorted square planar structure, with O1—Cu1—N1, O1A—Cu1—O1 and O1—Cu1—N1A angles of 91.24 (7) °, 180 ° and 88.76 (7) °, respectively [symmetry code: (A) = -x+1, -y+1, -z+1]. The Cu1—O1 and Cu1—N1 bond lengths are 1.9076 (16) and 2.005 (2) Å, respectively.

In the crystal, molecules are linked *via* Cu1···Cl^i^ interactions with a distance of 2.9933 (11) Å [symmetry code: (i) x, -y+0.5, z-0.5] which results in the formation of a two-dimensional network parallel to the bc plane (Fig. 2). There are also Cl1···Cl2^ii^ interactions present involving adjacent molecules [symmetry code: (ii) x+1, y, z+1] with distance 3.3709 (14) Å, which consolidate the two-dimensional network structure.

### Experimental

To an methanol solution (10 ml) containing 4-chlorobenzenamine (0.2 mmol, 25.4 mg) and 3-bromo-5-chlorosalicylaldehyde (0.2 mmol, 47.2 mg) was added CuCl_2_.2H_2_O (0.1 mmol, 17.1 mg) in methanol (10 ml). The mixture was stirred for 30 min and then filtered. The filtrate was left to stand undisturbed at room temperature. Dark-green prism-like crystals of the title complex was obtained by slow evaporation of the methanol solvent.

### Refinement

The C bound H atoms were included in calculated positions and treated as riding atoms: C—H = 0.93 Å with *U*_iso_(H) = 1.2*_U_*_eq_(C).

### Crystal data

**Table:**

[Cu(C_13_H_7_BrCl_2_NO)_2_]	*F*(000) = 734
*M**_r_* = 751.55	*D*_x_ = 1.904 Mg m^−^^3^
Monoclinic, *P*2_1_/*c*	Mo *K*α radiation, λ = 0.71073 Å
Hall symbol: -P 2ybc	Cell parameters from 4339 reflections
*a* = 11.064 (3) Å	θ = 2.1–29.1°
*b* = 9.437 (2) Å	µ = 4.32 mm^−^^1^
*c* = 13.277 (4) Å	*T* = 153 K
β = 108.997 (3)°	Block, green
*V* = 1310.8 (6) Å^3^	0.46 × 0.42 × 0.42 mm
*Z* = 2	

### Data collection

**Table:**

Rigaku AFC10/Saturn724+ diffractometer	3491 independent reflections
Radiation source: Rotating Anode	2777 reflections with *I* > 2σ(*I*)
Graphite monochromator	*R*_int_ = 0.033
Detector resolution: 28.5714 pixels mm^-1^	θ_max_ = 29.1°, θ_min_ = 2.7°
phi and ω scans	*h* = −13→15
Absorption correction: multi-scan (*CrystalClear*; Rigaku, 2008)	*k* = −9→12
*T*_min_ = 0.242, *T*_max_ = 0.265	*l* = −18→18
10994 measured reflections	

### Refinement

**Table:**

Refinement on *F*^2^	Primary atom site location: structure-invariant direct methods
Least-squares matrix: full	Secondary atom site location: difference Fourier map
*R*[*F*^2^ > 2σ(*F*^2^)] = 0.028	Hydrogen site location: inferred from neighbouring sites
*wR*(*F*^2^) = 0.065	H-atom parameters constrained
*S* = 1.00	*w* = 1/[σ^2^(*F*_o_^2^) + (0.031*P*)^2^ + 0.160*P*] where *P* = (*F*_o_^2^ + 2*F*_c_^2^)/3
3491 reflections	(Δ/σ)_max_ = 0.001
169 parameters	Δρ_max_ = 0.53 e Å^−^^3^
0 restraints	Δρ_min_ = −0.49 e Å^−^^3^

### Special details

**Table:**

Geometry. Bond distances, angles etc. have been calculated using the rounded fractional coordinates. All su's are estimated from the variances of the (full) variance-covariance matrix. The cell esds are taken into account in the estimation of distances, angles and torsion angles
Refinement. Refinement of *F*^2^ against ALL reflections. The weighted *R*-factor *wR* and goodness of fit *S* are based on *F*^2^, conventional *R*-factors *R* are based on *F*, with *F* set to zero for negative *F*^2^. The threshold expression of *F*^2^ > σ(*F*^2^) is used only for calculating *R*-factors(gt) *etc*. and is not relevant to the choice of reflections for refinement. *R*-factors based on *F*^2^ are statistically about twice as large as those based on *F*, and *R*- factors based on ALL data will be even larger.

### Fractional atomic coordinates and isotropic or equivalent isotropic displacement parameters (Å^2^)

**Table:**

	*x*	*y*	*z*	*U*_iso_*/*U*_eq_	
Br1	0.84869 (2)	0.57837 (3)	0.82867 (2)	0.0231 (1)	
Cu1	0.50000	0.50000	0.50000	0.0154 (1)	
Cl1	0.56447 (5)	0.30682 (6)	1.04373 (4)	0.0204 (2)	
Cl2	−0.17769 (6)	0.39010 (9)	0.25396 (6)	0.0444 (2)	
O1	0.60785 (15)	0.52907 (17)	0.64325 (12)	0.0175 (5)	
N1	0.35254 (17)	0.4484 (2)	0.54915 (14)	0.0159 (5)	
C1	0.5950 (2)	0.4776 (2)	0.73017 (17)	0.0148 (6)	
C2	0.6964 (2)	0.4879 (2)	0.82837 (18)	0.0170 (7)	
C3	0.6887 (2)	0.4339 (2)	0.92251 (17)	0.0178 (6)	
C4	0.5768 (2)	0.3680 (2)	0.92269 (17)	0.0178 (7)	
C5	0.4741 (2)	0.3556 (2)	0.83107 (17)	0.0180 (7)	
C6	0.4826 (2)	0.4100 (2)	0.73471 (17)	0.0165 (7)	
C7	0.3676 (2)	0.4035 (2)	0.64418 (18)	0.0177 (7)	
C8	0.2249 (2)	0.4343 (2)	0.47615 (17)	0.0175 (6)	
C9	0.1727 (2)	0.3013 (3)	0.44708 (18)	0.0218 (7)	
C10	0.0474 (2)	0.2874 (3)	0.38026 (19)	0.0254 (8)	
C11	−0.0229 (2)	0.4077 (3)	0.34322 (19)	0.0247 (7)	
C12	0.0273 (2)	0.5410 (3)	0.3717 (2)	0.0263 (8)	
C13	0.1522 (2)	0.5547 (3)	0.43829 (19)	0.0219 (7)	
H3	0.75910	0.44180	0.98640	0.0210*	
H5	0.39790	0.31070	0.83260	0.0220*	
H7	0.29510	0.36170	0.65570	0.0210*	
H9	0.22280	0.21920	0.47300	0.0260*	
H10	0.01090	0.19630	0.36050	0.0300*	
H12	−0.02350	0.62270	0.34580	0.0320*	
H13	0.18810	0.64610	0.45800	0.0260*	

### Atomic displacement parameters (Å^2^)

**Table:**

	*U*^11^	*U*^22^	*U*^33^	*U*^12^	*U*^13^	*U*^23^
Br1	0.0173 (1)	0.0301 (2)	0.0214 (1)	−0.0056 (1)	0.0054 (1)	−0.0027 (1)
Cu1	0.0132 (2)	0.0197 (2)	0.0126 (2)	−0.0016 (2)	0.0033 (1)	0.0003 (2)
Cl1	0.0253 (3)	0.0211 (3)	0.0134 (2)	−0.0044 (2)	0.0045 (2)	0.0031 (2)
Cl2	0.0161 (3)	0.0692 (5)	0.0384 (4)	−0.0005 (3)	−0.0041 (3)	−0.0151 (4)
O1	0.0160 (8)	0.0240 (9)	0.0119 (7)	−0.0035 (7)	0.0038 (6)	0.0001 (6)
N1	0.0124 (9)	0.0185 (10)	0.0163 (9)	−0.0004 (7)	0.0041 (7)	0.0005 (7)
C1	0.0163 (10)	0.0123 (11)	0.0157 (11)	0.0000 (8)	0.0051 (9)	−0.0008 (8)
C2	0.0168 (11)	0.0155 (12)	0.0187 (11)	−0.0007 (9)	0.0056 (9)	−0.0023 (9)
C3	0.0176 (11)	0.0172 (12)	0.0156 (10)	0.0007 (9)	0.0015 (9)	−0.0011 (9)
C4	0.0221 (12)	0.0156 (12)	0.0148 (10)	0.0007 (9)	0.0047 (9)	0.0013 (9)
C5	0.0187 (11)	0.0179 (12)	0.0168 (11)	−0.0023 (9)	0.0051 (9)	0.0004 (9)
C6	0.0161 (11)	0.0169 (12)	0.0149 (11)	0.0004 (9)	0.0029 (9)	0.0001 (8)
C7	0.0166 (11)	0.0181 (12)	0.0183 (11)	−0.0019 (9)	0.0056 (9)	−0.0006 (9)
C8	0.0139 (10)	0.0251 (13)	0.0140 (10)	−0.0019 (9)	0.0052 (9)	0.0000 (9)
C9	0.0199 (12)	0.0233 (13)	0.0204 (12)	−0.0033 (10)	0.0040 (10)	0.0019 (9)
C10	0.0246 (13)	0.0267 (14)	0.0237 (12)	−0.0097 (11)	0.0062 (10)	−0.0032 (10)
C11	0.0114 (11)	0.0438 (16)	0.0174 (11)	−0.0013 (10)	0.0027 (9)	−0.0051 (10)
C12	0.0179 (12)	0.0345 (15)	0.0250 (13)	0.0084 (11)	0.0050 (10)	−0.0012 (11)
C13	0.0203 (12)	0.0219 (13)	0.0232 (12)	0.0004 (10)	0.0066 (10)	0.0000 (10)

### Geometric parameters (Å, º)

**Table:**

Br1—C2	1.888 (2)	C4—C5	1.373 (3)
Cu1—O1	1.9076 (16)	C5—C6	1.410 (3)
Cu1—N1	2.005 (2)	C6—C7	1.439 (3)
Cu1—Cl1^i^	2.9933 (11)	C8—C9	1.383 (3)
Cu1—O1^ii^	1.9076 (16)	C8—C13	1.388 (3)
Cu1—N1^ii^	2.005 (2)	C9—C10	1.388 (3)
Cu1—Cl1^iii^	2.9933 (11)	C10—C11	1.374 (4)
Cl1—C4	1.754 (2)	C11—C12	1.377 (4)
Cl2—C11	1.745 (3)	C12—C13	1.383 (3)
O1—C1	1.302 (3)	C3—H3	0.9500
N1—C7	1.289 (3)	C5—H5	0.9500
N1—C8	1.435 (3)	C7—H7	0.9500
C1—C2	1.420 (3)	C9—H9	0.9500
C1—C6	1.416 (3)	C10—H10	0.9500
C2—C3	1.378 (3)	C12—H12	0.9500
C3—C4	1.386 (3)	C13—H13	0.9500
			
O1—Cu1—N1	91.24 (7)	C4—C5—C6	119.5 (2)
Cl1^i^—Cu1—O1	94.96 (5)	C1—C6—C5	121.4 (2)
O1—Cu1—O1^ii^	180.00	C1—C6—C7	122.24 (19)
O1—Cu1—N1^ii^	88.76 (7)	C5—C6—C7	116.1 (2)
Cl1^iii^—Cu1—O1	85.04 (5)	N1—C7—C6	126.9 (2)
Cl1^i^—Cu1—N1	97.49 (6)	N1—C8—C9	120.15 (19)
O1^ii^—Cu1—N1	88.76 (7)	N1—C8—C13	119.69 (19)
N1—Cu1—N1^ii^	180.00	C9—C8—C13	120.1 (2)
Cl1^iii^—Cu1—N1	82.51 (6)	C8—C9—C10	120.3 (2)
Cl1^i^—Cu1—O1^ii^	85.04 (5)	C9—C10—C11	118.8 (3)
Cl1^i^—Cu1—N1^ii^	82.51 (6)	Cl2—C11—C10	118.7 (2)
Cl1^i^—Cu1—Cl1^iii^	180.00	Cl2—C11—C12	119.5 (2)
O1^ii^—Cu1—N1^ii^	91.24 (7)	C10—C11—C12	121.7 (2)
Cl1^iii^—Cu1—O1^ii^	94.96 (5)	C11—C12—C13	119.4 (2)
Cl1^iii^—Cu1—N1^ii^	97.49 (6)	C8—C13—C12	119.7 (2)
Cu1^iv^—Cl1—C4	103.02 (7)	C2—C3—H3	120.00
Cu1—O1—C1	128.08 (15)	C4—C3—H3	120.00
Cu1—N1—C7	122.60 (16)	C4—C5—H5	120.00
Cu1—N1—C8	121.93 (14)	C6—C5—H5	120.00
C7—N1—C8	114.56 (19)	N1—C7—H7	117.00
O1—C1—C2	120.5 (2)	C6—C7—H7	117.00
O1—C1—C6	123.8 (2)	C8—C9—H9	120.00
C2—C1—C6	115.73 (19)	C10—C9—H9	120.00
Br1—C2—C1	118.09 (16)	C9—C10—H10	121.00
Br1—C2—C3	118.95 (17)	C11—C10—H10	121.00
C1—C2—C3	123.0 (2)	C11—C12—H12	120.00
C2—C3—C4	119.1 (2)	C13—C12—H12	120.00
Cl1—C4—C3	119.02 (17)	C8—C13—H13	120.00
Cl1—C4—C5	119.67 (17)	C12—C13—H13	120.00
C3—C4—C5	121.3 (2)		
			
N1—Cu1—O1—C1	22.33 (18)	C6—C1—C2—C3	1.2 (3)
Cl1^i^—Cu1—O1—C1	119.96 (17)	O1—C1—C6—C5	179.73 (19)
N1^ii^—Cu1—O1—C1	−157.67 (18)	O1—C1—C6—C7	−5.7 (3)
Cl1^iii^—Cu1—O1—C1	−60.04 (17)	C2—C1—C6—C5	−0.7 (3)
O1—Cu1—N1—C7	−22.22 (18)	C2—C1—C6—C7	173.92 (19)
O1—Cu1—N1—C8	169.30 (16)	Br1—C2—C3—C4	179.69 (15)
Cl1^i^—Cu1—N1—C7	−117.39 (17)	C1—C2—C3—C4	−0.9 (3)
Cl1^i^—Cu1—N1—C8	74.13 (15)	C2—C3—C4—Cl1	−177.01 (16)
O1^ii^—Cu1—N1—C7	157.78 (18)	C2—C3—C4—C5	0.1 (3)
O1^ii^—Cu1—N1—C8	−10.70 (16)	Cl1—C4—C5—C6	177.47 (15)
Cl1^iii^—Cu1—N1—C7	62.61 (17)	C3—C4—C5—C6	0.4 (3)
Cl1^iii^—Cu1—N1—C8	−105.88 (15)	C4—C5—C6—C1	−0.1 (3)
Cu1^iv^—Cl1—C4—C3	−124.36 (15)	C4—C5—C6—C7	−174.99 (18)
Cu1^iv^—Cl1—C4—C5	58.52 (17)	C1—C6—C7—N1	4.1 (3)
Cu1—O1—C1—C2	167.99 (14)	C5—C6—C7—N1	178.9 (2)
Cu1—O1—C1—C6	−12.4 (3)	N1—C8—C9—C10	177.1 (2)
Cu1—N1—C7—C6	13.8 (3)	C13—C8—C9—C10	−0.2 (3)
C8—N1—C7—C6	−176.95 (19)	N1—C8—C13—C12	−177.1 (2)
Cu1—N1—C8—C9	103.7 (2)	C9—C8—C13—C12	0.2 (4)
Cu1—N1—C8—C13	−78.9 (2)	C8—C9—C10—C11	0.5 (3)
C7—N1—C8—C9	−65.6 (3)	C9—C10—C11—Cl2	176.83 (18)
C7—N1—C8—C13	111.7 (2)	C9—C10—C11—C12	−0.8 (4)
O1—C1—C2—Br1	0.2 (3)	Cl2—C11—C12—C13	−176.80 (19)
O1—C1—C2—C3	−179.21 (19)	C10—C11—C12—C13	0.8 (4)
C6—C1—C2—Br1	−179.41 (14)	C11—C12—C13—C8	−0.5 (4)

Symmetry codes: (i) −*x*+1, *y*+1/2, −*z*+3/2; (ii) −*x*+1, −*y*+1, −*z*+1; (iii) *x*, −*y*+1/2, *z*−1/2; (iv) −*x*+1, *y*−1/2, −*z*+3/2.
